# Salvage esophagectomy for unresectable locally advanced esophageal squamous cell carcinoma: Significant or not?

**DOI:** 10.1002/ags3.70013

**Published:** 2025-04-21

**Authors:** Hiroshi Saeki, Makoto Sakai, Takayoshi Watanabe, Makoto Sohda, Ken Shirabe

**Affiliations:** ^1^ Department of General Surgical Science Gunma University Graduate School of Medicine Maebashi Gunma Japan; ^2^ Department of Surgery Japanese Red Cross Maebashi Hospital Maebashi Gunma Japan; ^3^ Department of Gastroenterological Surgery Gunma Prefectural Cancer Center Ota Gunma Japan

**Keywords:** advanced esophageal cancer, definitive chemoradiotherapy, immunotherapy, minimally invasive surgery, T classification

## Abstract

We reviewed the current status and perspectives on salvage esophagectomy for initially unresectable locally advanced esophageal squamous cell carcinoma (ESCC) in the era of minimally invasive surgery and immunotherapy. Although the standard treatment for these patients is definitive chemoradiotherapy (CRT), the complete response rate to CRT alone remains unsatisfactory. Salvage esophagectomy, which is defined as surgery for residual or recurrent lesions after definitive CRT, is considered a curative treatment in clinical practice. No randomized trials have been conducted comparing salvage esophagectomy and non‐surgical treatment in this cohort, because, in addition to the small number of eligible patients, constructing an appropriate study design may have been difficult. Therefore, in this review, the assessment of the current status was based on the results of several available retrospective studies. Most results from these studies show favorable results for salvage esophagectomy in this subject; however, whether it is a widely used treatment should be carefully evaluated because all these reports are limited to those from high‐volume facilities for esophageal surgery. Appropriate patient selection and skilled surgical techniques are essential for successful salvage esophagectomy for initially unresectable locally advanced ESCC. To improve the short‐ and long‐term outcomes of this surgery, advances in surgical techniques as well as further development of diagnostic capabilities, perioperative management, and multidisciplinary treatment are desirable.

## 1 INTRODUCTION

Treatment strategies for initially unresectable, locally advanced esophageal squamous cell carcinoma (ESCC) require further development. Definitive chemoradiotherapy (CRT) is the standard treatment option for patients without distant metastases. However, the cure rate for unresectable locally advanced ESCC is still unsatisfied.^1^, ^2^, ^3^, ^4^ Endoscopic submucosal dissection and photodynamic therapy, which are treatments for salvage purposes, are mainly indicated for superficial cancers.^5^, ^6^, ^7^ Therefore, salvage esophagectomy is the only potentially curative treatment for residual or recurrent disease after definitive CRT for locally advanced cancer.^8^, ^9^ When considering salvage esophagectomy for locally advanced ESCC, important issues are the curability and safety of the surgery. Factors related to these include the difficulty of preoperative diagnosis whether cT4 tumors have been down‐staged to be resectable, the high invasiveness of the surgery itself, the difficulty of surgical techniques such as dissection of invasive areas to other organs, the lack of established surgical procedures such as the extent of lymph node dissection, and the decline in the patient's general condition due to definitive CRT.

On the other hand, the outcomes of esophageal cancer surgery have generally improved over the past years due to improved diagnostic accuracy and perioperative management, advances in multidisciplinary treatment, and the popularization of minimally invasive surgery (i.e., the widespread use of thoracoscopic and robotic surgeries).^10^, ^11^, ^12^, ^13^, ^14^, ^15^ In recent years, immunotherapy has become an essential modality for the treatment of ESCC, and is expected to be effective even against locally advanced cancers. However, whether these advances in esophageal cancer treatment are directly applicable to salvage esophagectomy for unresectable locally advanced ESCC is unclear. Importantly, the number of applicable cases is not large enough to conduct large‐scale prospective studies, making it difficult to establish high‐level evidence in this field.

Based on the above background, reviewing the current real‐world position of salvage esophagectomy for unresectable locally advanced ESCC would be valuable with updated findings in the literature. We hope that this review contributes to the development of treatments for initially unresectable locally advanced ESCC.

## 2 DIAGNOSIS OF cT4

In imaging studies, clarifying the diagnostic criteria is necessary for cT4 tumors. The involvement of the adjacent organs is generally determined using CT. Tumors are classified as cT4 if they extend into the lumen, cause airway deformity, or are attached to the aorta at a contact angle of >90*°* in CT slices.^16^ Several clinical studies have adopted this diagnostic criteria.^17^, ^18^ However, inter‐observer variations are observed in the clinical diagnosis of cT4 ESCC.^19^ In the 12th edition of the Japanese Classification of Esophageal Cancer, cT3 is newly sub‐classified as either resectable (cT3r) or borderline resectable (cT3br).^20^ When the suspicious target organs of invasion are the trachea, bronchus, or aorta, which are usually not resected simultaneously, but no definite cT4 findings are found, the tumor is classified as cT3br. Although cT3br was defined as the possibility of considering treatment strategies aimed at curative surgery, some patients may be candidates for salvage esophagectomy.^21^, ^22^ No clear imaging definition exists for cT3r/T3br/T4, and its lack of objectivity requires improvement. We believe that such a classification would be useful when considering treatment strategies. Diagnostic modalities other than CT, such as magnetic resonance imaging and endobronchial ultrasound, have been also expected.^23^, ^24^ Improvements in the imaging diagnostics of invasion to other organs will undoubtedly play an important role in the success of salvage esophagectomy.

## 3 GUIDELINES FOR TREATMENT OF cT4 ESCC

In the esophageal cancer practice guidelines 2022, edited by the Japan esophageal society,^1^ definitive CRT is weakly recommended as a treatment for cT4 ESCC without distant metastasis (i.e., cStage IVA). In Japan, based on the results of the JCOG0303 study, definitive CRT is usually selected for the treatment of patients with unresectable locally advanced ESCC.^17^ We conducted a search of scientific literature on PubMed/MEDLINE and Web of Science to obtain all relevant articles published from January 2000 to December 2024. In the searches, we excluded all non‐English articles. The search terms were “esophageal cancer,” “locally advanced,” “T4,” and “chemoradiotherapy.” Articles were selected when the abstract indicated that data were collected on patients who received definitive CRT (dCRT) for unresectable locally advanced (T4), thoracic ESCC included in randomized controlled trials or prospective phase II studies. We also reviewed the reference lists of these articles to find additional candidate studies. In this chapter, we excluded articles that included immunotherapy.

According to the literature obtained by the above methods, the 2‐ or 3‐year survival rate was reported to be in the range of about 20%–40% (Table 1).^17^, ^25^, ^26^, ^27^, ^28^, ^29^, ^30^ The chemotherapy regimen used in these studies were mainly 5‐FU + cisplatin. Conversion surgery after induction therapy for cT4 ESCC cases has received much attention, and relatively high curative resection rates have been reported.^31^ However, the difference in outcomes between conversion surgery after induction chemotherapy and salvage surgery after dCRT has not been clarified. More recently, a treatment modality has been developed for patients with unresectable locally advanced ESCC, which involves intense induction chemotherapy with docetaxel plus cisplatin and 5‐fluorouracil, followed by curative surgery.^32^, ^33^, ^34^, ^35^ A phase II trial reported promising results, with a median overall survival of approximately 34 months and a 0% treatment‐related mortality rate,^36^ and a Phase III study of tri‐modality combination therapy with induction docetaxel plus cisplatin and 5‐fluorouracil versus definitive CRT for locally advanced unresectable ESCC (JCOG1510)^37^ is ongoing.

**TABLE 1:** Prospective studies on definitive chemoradiotherapy for unresectable locally advanced esophageal squamous cell carcinoma.

Author	Year	Study name/phase	Number of cases	Radiation dose (Gy)	Chemotherapy regimen	CR rate	Mortality rate	Survival rate
Nishimura et al.^25^	2002	‐/‐	25	60	CF	32	7	23% at 2y‐OS
Ishida et al.^26^	2004	JCOG9516/II	60	60	CF	15	3	32% at 2y‐OS
Tomblyn et al.^27^	2012	SWOG (S0414)/II	21	50.4	Cet/C/Iri	6	10	33% at 2y‐OS
Higuchi et al.^28^	2014	KDOG 0501–P2/II	42	50.4‐61.2	DCF	30/60^a^	2	44% at 3y‐OS
Shinoda et al.^17^	2015	JCOG0303/II, III	142	60	CF (Arm A: low dose; Arm B: standard)	Arm A: 0	Arm A: 3	Arm A: 26% at 3y‐OS
						Arm B: 1	Arm B: 1	Arm B: 26% at 3y‐OS
Satake et al.^29^	2016	‐/I, II	33	60	Induction DCF concurrent CF	39	0	40% at 3y‐OS
Hihara et al.^30^	2016	‐/II	11	66	DF	31	0	31% at 5y‐OS

Abbreviations: C, CDDP; Cet, cetuximab; D, docetaxel; F, 5‐FU; Iri, irinotecan.

^a^ Indicated for 50.4/61.2 Gy.

In the ESMO clinical practice guidelines, treatment with definitive CRT is recommended for patients with ESCC or esophageal adenocarcinoma that is unresectable and locally advanced, or those who are unable or unwilling to undergo surgery.^3^ Salvage esophagectomy is mentioned only for resectable locally advanced cancer, and not for initially unresectable disease. In the NCCN Guidelines of Esophageal and Esophagogastric Junction Cancers, Version 5, 2024 (https://www.nccn.org/professionals/physician_gls/pdf/esophageal.pdf), definitive CRT is recommended as the primary treatment for patients with cT4 ESCC who are medically fit, whereas chemotherapy alone is considerable in the setting of invasion of the trachea, great vessels, vertebral body, or heart. Esophagectomy is a management strategy for persistent local disease.

## 4 SALVAGE ESOPHAGECTOMY FOR UNRESECTABLE LOCALLY ADVANCED ESCC

According to the Esophageal Cancer Practice guidelines 2022,^1^ surgical resection for patients with unresectable locally advanced ESCC that becomes resectable after definitive CRT or induction chemotherapy is weakly recommended (consensus rate: 89.3% [25/28]; evidence level: C). To date, no randomized trials have compared surgery with non‐surgery in this cohort. In the JCOG0303 study, 16.9% of patients (12 of 71 patients) underwent surgery for residual or recurrent lesions after completion of the protocol‐specified treatment.^17^

We conducted a search of scientific literature on PubMed/MEDLINE and Web of Science to obtain all relevant articles published from January 2000 to December 2024. In the searches, we excluded all non‐English articles and reports with fewer than 10 cases. The search terms were “salvage esophagectomy,” “chemoradiotherapy,” “locally advanced,” “T4,” and “esophageal cancer.” Articles were selected when the abstract indicated that data were collected on patients who underwent salvage esophagectomy for initially unresectable, locally advanced (T4), thoracic ESCC included in retrospective studies or randomized controlled trials. We also reviewed the reference lists of these articles to find additional candidate studies. The extracted reports are summarized in the Table 2.^18^, ^33^, ^38^, ^39^, ^40^, ^41^, ^42^, ^43^, ^44^ Sample sizes ranged from 12 to 72. Radiation doses and chemotherapy regimens varied among the studies. R0 resection rate of 42%–92%. The incidences of anastomotic leakage and pneumonia were in 6%–39% and 6%–46%, respectively. The mortality rate was 0%–10%. Patients who underwent salvage surgery had a 5‐year overall survival rate of 6%–52%. The article by Defize et al. is of interest because they reported the results of salvage robot‐assisted minimally invasive esophagectomy (MIE) performed for all patients with T4b esophageal cancer.^44^ No conversions or major intraoperative complications were observed in all 24 patients. Curative resection was achieved in 22 patients (92%), and the disease‐free survival at 24 months was 68%.

**TABLE 2:** Studies of salvage esophagectomy for initially unresectable locally advanced esophageal squamous cell carcinoma.

Author	Year	Number of cases	Radiation dose (Gy)	Chemotherapy regimen	MIE rate	R0 resection rate	Postoperative complication rate	Mortality rate	Survival rate
^a^Miyata et al.^39^	2019	72	40–60	CF	0	89	AL: 8	1	43% at 5y‐OS
							PC: 13		
Ohkura et al.^33^	2019	33	50.4–71.4	DCF/CF	9	42	AL: 12	0	91% at 5y‐OS (R0)
							Chylothorax: 6		0% at 5y‐OS (R1/2)
Hashimoto et al.^40^	2020	12	49.6–60	DCF	8	92	AL: 25	0	50% at 3y‐OS
							Pneumonia: 25		
Booka et al.^41^	2020	18	60	CF	0	78	AL: 39	0	52% at 5y‐OS
							Pneumonia: 6		
Okamura et al.^42^	2020	35	50–70	CF	‐	54	AL: 6	9	6% at 5y‐OS
							Pneumonia: 11		
Harada et al.^43^	2020	15	50.4	DCF/CF/NF	0	73	AL: 27	0	82% at 5y‐OS (R0)
							PC: 0		25% at 5y‐OS (R1)
Sugawara et al.^18^	2020	31	50.4–65.4	F or S with C or N	0	71	AL: 16	10	59% at 3y‐OS (R0)
							Pneumonia: 29		0% at 3y‐OS (R1/2)
^b^Sugimura et al.^35^	2021	49	50.4	CF		78	AL: 13	0	N/A
							Pneumonia:18		
Defize et al.^44^	2021	24	50.4	Car Pac	100	91.7	AL: 25	4	51% at 2y‐OS
							Pneumonia: 46		

Abbreviations: AL, anastomotic leakage; C, CDDP; Car, Carboplatin; D, docetaxel; F, 5‐FU; F, nedaplatin; MIE, minimally invasive esophagectomy; N/A, not available; OS, overall survival; Pac, paclitaxel; PC, pulmonary complication; S, S‐1.

^a^ These 72 cases include some induction chemotherapy cases.

^b^ Data of chemoradiotherapy arm from randomized study are shown.

The overall outcomes after salvage esophagectomy for locally advanced ESCC varied among the studies, showing that the patient selection criteria and surgical procedures were not consistent among the facilities. In some studies,^18^, ^39^, ^43^ survival after salvage esophagectomy was compared between R0 and non‐R0 patients. All of these studies indicated that patients who underwent R0 resection had extremely favorable long‐term survival. However, it is difficult to determine preoperatively whether R0 resection is feasible. Some previous studies have suggested the predictive factors for R0 resection.^45^, ^46^ Although there is a report on cases with good indications for salvage surgery,^47^ the clinical decision remains difficult to make in practice.

For patients with cT4 disease, performing salvage surgery after dCRT likely requires a certain level of response to dCRT. On the other hand, in cases where a good response is observed, an active surveillance strategy could also be considered. Since reliable diagnostic criteria for clinical response has not been established in clinical practice,^48^, ^49^ the relatively aggressive strategy deems reasonable for patients with remnant or recurrent ESCC after dCRT.^49^ However, some studies have shown that patients with a good response to preoperative CRT obtain no survival benefit by subsequent surgical treatment.^50^, ^51^, ^52^ Active surveillance strategy can be useful therapeutic alternative for those patients.^53^

In salvage surgery, optimization of the extent of lymph node dissection is crucial for improving patient's outcomes. Surgeons often perform selective lymph node dissection, such as omitting the subcarinal dissection of lymph node, in order to preserve tracheal blood flow.^54^ This approach is reportedly useful for reducing fatal tracheal and pulmonary problems in patients who underwent salvage surgery.^54^, ^55^, ^56^ However, considering that residual or recurrent ESCC after dCRT is biologically aggressive, extensive lymph node dissection might have survival benefits for patients. In fact, some investigators have suggested the benefits of prophylactic mediastinal lymph node dissection.^39^, ^57^

## 5 MINIMALLY INVASIVE SALVAGE ESOPHAGECTOMY

Some reports have described MIE, including thoracoscopic and robotic surgeries, indicated for salvage esophagectomy. Abe et al. reported the clinical results of 31 thoracoscopic salvage esophagectomies performed using prophylactic mediastinal lymph node dissection.^57^ They compared with 610 non‐salvage patients who underwent conventional thoracoscopic esophagectomy, and concluded that thoracoscopic salvage esophagectomy was as safe as conventional thoracoscopic esophagectomy. Among the 31 patients who underwent thoracoscopic salvage esophagectomy, eight (26%) had cT4 disease. It was reported that postoperative pneumonia after salvage esophagectomy worsened the outcomes of patients.^58^ Therefore, strategies to reduce postoperative complications, including pulmonary problems, are important when performing salvage esophagectomy. Ishiyama et al. investigated 82 patients who underwent salvage esophagectomy for thoracic esophageal cancer and compared perioperative factors and postoperative complications between the open (*n* = 62) and thoracoscopy groups (*n* = 20).^56^ In multivariate analysis, thoracoscopic surgery was an independent favorable factor associated with postoperative pneumonia (odds ratio: 0.29; *p* = 0.04) and total complications (odds ratio: 0.26; *p* = 0.02). Seven cT3‐cT4 patients (35%) were included in the thoracoscopy group; however, cT3 and cT4 were not distinguished in this study. As detailed above, Defize et al. focused on salvage robot‐assisted MIE for T4b esophageal cancer, which is the only report to date showing the results of robotic surgery in this cohort.^44^

All three reports demonstrated the usefulness of MIE in salvage esophagectomy in initially unresectable cases. High‐definition video systems for thoracoscopic and robotic surgeries have advantages over open surgery in that they provide a clear image of the microanatomical structure. Advances in imaging technology have enabled surgeons to recognize layers of dissection that are invisible during open surgery under a magnified field of view. Additionally, an increased understanding of the microanatomical structures within the tracheal wall through lateral longitudinal anastomosis provided by MIE may prevent tracheal ischemia.

However, these previous reports were all based on results from high‐volume esophageal surgery centers. The relationship between the number of surgical cases and outcomes has been extensively reported for esophagectomies.^59^, ^60^ Further centralization of this complex surgery could further enhance outcomes.

## 6 IMMUNOTHERAPY FOR LOCALLY ADVANCED ESCC

Recently, immunotherapy has become the central treatment modality for advanced and recurrent esophageal cancers. Based on the results of randomized phase III trials, KEYNOTE‐590 and CheckMate 648, immune checkpoint inhibitors (ICIs) combined with chemotherapy or double ICIs have been recommended as first‐line treatments instead of chemotherapy alone for advanced or recurrent esophageal cancer.^61^, ^62^ It is not yet clear how effective ICI ± chemotherapy is for unresectable locally advanced ESCC and how much conversion surgery can be expected.

Radiation triggers DNA damage‐induced cell death in cancer cells and also alter tumor antigenicity. This is due to activation of cytoplasmic DNA sensors, induction of immunogenic cell death, enhancement of neoantigen expression, and modulation of the tumor microenvironment.^63^, ^64^ Thus, combining immuno‐oncology approaches with radiation can enhance the response to ICI in cancers.

Whether combined immunotherapy with definitive CRT improves the therapeutic efficacy for unresectable locally advanced ESCC remains unclear.^65^ A phase II clinical trial evaluated the efficacy of durvalumab and tremelimumab in conjunction with definitive CRT (5‐FU + cisplatin) in patients with unresectable locally advanced ESCC.^66^ In another phase Ib trial, camrelizumab in combination with apatinib and concurrent CRT (docetaxel + cisplatin) was also evaluated.^67^ These two small prospective studies suggested that the combination of immunotherapy and definitive CRT could improve survival compared with traditional definitive CRT. To date, several phase III randomized multicenter trials on definitive CRT with concurrent or sequential ICI for locally advanced ESCC are ongoing (Table 3).^68^, ^69^ The combination of these novel treatment strategies with salvage esophagectomy may be an issue for therapeutic development over the next decade.

**TABLE 3:** Immunotherapy combined with definitive chemoradiotherapy for unresectable esophageal squamous cell carcinoma: Ongoing phase III trials.

Study name (ID)	Histological type	Control arm	Study arm	Primary endpoints	Secondary endpoints
RATIONALE‐311^50^ (NCT03957590)	ESCC	Placebo + CRT	Tislelizumab + CRT	PFS	ORR, DoR, OS, AEs
KEYNOTE‐975^51^ (NCT04210115)	ESCC/EAC/GEJC	Placebo + CRT	Pembrolizumab + CRT	OS, EFS	AEs
KUNLUN (NCT04550260)	ESCC	Placebo + CRT	Durvalumab + CRT	PFS	OS, AEs
ESCORT‐CRT (NCT04426955)	ESCC	Placebo + CRT	Camrelizumab + CRT	PFS	OS, ORR, DoR, AEs
SKYSCRAPER‐07 (NCT045436)^17^	ESCC	CRT → Tiragolumab	Arm A: CRT → Tiragolumab + Atezolizumab	PFS, OS	ORR, DoR, QoL, AEs
		Placebo + Atezolizumab			
		Placebo	Arm B: CRT → Tiragolumab		
			Placebo + Atezolizumab		

Abbreviations: AEs, adverse events; CRT, chemoradiotherapy; DoR, duration of response; EAC, esophageal adenocarcinoma; EFS, event‐free survival; ESCC, esophageal squamous cell carcinoma; GEJC, gastroesophageal junction cancer; ORR, objective response rate; OS, overall survival; PFS, progression‐free survival; QoL, quality of life.

## 7 DISCUSSION

In this review, we summarized the literature on salvage esophagectomy for unresectable locally advanced ESCC. Several retrospective studies have reported acceptable or favorable clinical outcomes after surgery. However, it should be noted that all of these reports were published from specialized esophageal surgery centers, and these outcomes may not be achievable at every institution. Interestingly, most clinical results have been reported in Japanese institutions since 2019. This may indicate that esophageal surgeons in Japan consider relatively aggressive indications for salvage surgery, even in initially unresectable cases. In this context, it is noteworthy that a coherent set of robotic surgery results has been published in the Netherlands.^44^ The worldwide trend toward MIE may also have influenced this surgery.

Salvage esophagectomy is widely performed for cStage I–IV esophageal cancer. Among them, salvage esophagectomy for cT4 disease may be the most challenging. The conditions for successful salvage esophagectomy include achievement of R0 surgery and avoidance of serious postoperative complications. Several previous reports have indicated that serious complications worsen the long‐term prognosis of esophageal cancer.^70^, ^71^, ^72^ Comprehensive improvements in diagnosis, surgical techniques, perioperative management, and multidisciplinary treatments are required to achieve these conditions.

Ideally, salvage esophagectomy for unresectable locally advanced ESCC should be validated in well‐designed prospective randomized trials. However, in practice, conducting a trial is unrealistic due to the small number of eligible patients, limited number of facilities where surgery can be performed, and difficulty in conducting a trial because active surveillance or drug therapy alone for possible comparison is extremely different from salvage esophagectomy. Therefore, we conducted an ongoing nationwide survey of patients who had undergone salvage esophagectomy for cT4 thoracic ESCC at institutions accredited by the Japan Esophageal Society.

In conclusion, safe and effective salvage esophagectomy has been performed for unresectable locally advanced ESCC in a limited number of institutions. However, the general prevalence of this procedure should be carefully evaluated. We hope that many patients benefit from further clinical studies.

## AUTHOR CONTRIBUTIONS

**Hiroshi Saeki:** Writing – original draft; writing – review and editing. **Makoto Sakai:** Writing – review and editing. **Takayoshi Watanabe:** Writing – review and editing. **Makoto Sohda:** Writing – review and editing. **Ken Shirabe:** Supervision.

## 8 FUNDING INFORMATION

This study did not receive any specific grants from funding agencies in the public, commercial, or non‐profit sectors.

## CONFLICT OF INTEREST STATEMENT

Hiroshi Saeki and Ken Shirabe are the editorial members of *Annals of Gastroenterological Surgery*.

## ETHICS STATEMENT

Approval of the research protocol by an Institutional Reviewer Board: N/A.

Informed Consent: N/A.

Registry and the Registration No. of the study/trial: N/A.

Animal Studies: N/A.
